# Heat Treatment Influencing Porosity and Tensile Properties of Field Assisted Sintered AlSi7Mg0.6

**DOI:** 10.3390/ma15072503

**Published:** 2022-03-29

**Authors:** Sarah Johanna Hirsch, Lisa Winter, Thomas Grund, Thomas Lampke

**Affiliations:** Materials and Surface Engineering Group, Institute of Materials Science and Engineering, Chemnitz University of Technology, D-09125 Chemnitz, Germany; lisa.winter@mb.tu-chemnitz.de (L.W.); thomas.grund@mb.tu-chemnitz.de (T.G.); thomas.lampke@mb.tu-chemnitz.de (T.L.)

**Keywords:** field assisted sintering technique, A357 aluminum alloy, mechanical properties, microstructure, porosity

## Abstract

In this study, an attempt was made to improve the mechanical properties and in particular the strength of a precipitation-hardenable aluminum alloy while still maintaining high ductility. For this purpose, AlSi7Mg0.6 (A357) powder with an average particle diameter of d_50_ = 40 µm was consolidated using field assisted sintering technique (FAST), and two material conditions were compared: an as-sintered and an underaging heat treated condition (T61). Mechanical properties were determined using tensile tests and hardness measurements. In addition, the microstructure was investigated by optical microscopy. Further, porosity and density were analyzed after the different heat treatments. By the underaging heat treatment, the surface hardness was increased by 100% and the yield strength was increased by 80% compared to the as-sintered material. However, the elongation to failure dropped to one third of that of the as-sintered material. Presumably, this effect was a result of an increased porosity due to the heat treatment. It is assumed that the observed pores were generated by artefacts from the FAST process used to manufacture the samples. The internal gas pressure and equilibrium diffusion supported by heat treatment temperature, and the reduction in surface energy caused by coalescent micropores, led to the enlargement of previously undetectable inhomogeneities in the as-sintered material that resulted in pores in the heat-treated sintered alloy.

## 1. Introduction

A lot of research has already been done on improving the mechanical behavior of aluminum. However, the potential applications of cast aluminum alloys in safety-relevant fields of automobiles, for example, are not yet fully covered. Crash-relevant components are characterized by their demand for a high energy absorption capacity. Hence, these applications need materials that combine high plastic deformation capacity with high strength. Aluminum–silicon alloys are among the most widely used casting materials due to their high strength, good ductility [1,2] and high wear and corrosion resistance [1]. The mechanical properties of these casting alloys are mainly influenced by the microstructure formed during solidification, although the toughness can be adapted to the technical requirements by subsequent heat treatment to form the silicon lamellae [1].

Hypereutectic AlSi alloys with silicon content below the eutectic composition of 12.5 wt.% Si crystallize due to the decreasing solution of Si to dendritic α-solid solution grains. Simultaneously, the residual melt is enriched with Si until the eutectic composition is reached and crystallizes in a eutectic microstructure. The eutectic crystal phases appear in the form of lamellae, fibers or rods [1]. According to Shabestari and Moemeni [3], who investigated the effects of copper on the mechanical properties of a AlSi7Mg alloy, the refinement of this microstructure leads to significant improvements in mechanical properties. In addition to the chemical composition, melting process and casting process, the solidification rate determines the microstructure and hence the mechanical properties of aluminum parts. The spacing of the secondary dendrite arms controls the size and distribution of porosity and intermetallic particles, depending on the composition of the alloy in the casted material [3]. Cai et al. [4] added Mn instead of Cu to an AlSiMg casting alloy. For a 30%-eutectic alloy, they obtained fine Si phases, high yield strength and fracture strength and high ductility. Research on influencing the microstructure of Al–Si casting alloys also included the addition of modifiers and refiners [1,5,6,7,8] and mechanical post-treatment [9]. However, the cited methods only refer to the melt-metallurgical production route of AlSi materials and address a mostly inhomogeneous microstructure over the cross-section of the casted parts, which results from casting die geometries and unbalanced cooling rates. An alternative production route for similar AlSi materials can be followed with solid-state processes, also known as powder metallurgy, producing compact pieces whose microstructures can be influenced much more easily. Solid-state processes consolidate powders at temperatures below their melting ranges. The diffuse mass transport leads to bonding between the particles and ultimately to the formation of a dense piece. The advantages of powder metallurgy include, in addition to the saving of energy by working in the solid phase range, the near-net-shape production [10,11], the manufacturing of defined dense parts [10] and the almost unlimited variability of the initial microstructure through the use of powder particle fractions of different sizes. Due to the regular distribution of powder fraction in the sintering tool cavity, a regular microstructure is created throughout the entire component during consolidation. Using this strategy, the possible problem of cast structures; the gradual transition of properties and the directional microstructure due to coarse dendrites; and a heterogeneously distributed eutectic microstructure, can be avoided. As a consequence of fine, sintered microstructure, significant increases in strength and ductility can be achieved. Aluminum and its alloys are materials that are mainly predestined for melt metallurgical processes. However, it may be possible to generate improved mechanical properties even by powder metallurgical routes, such as the fast and robust field assisted sintering technique (FAST).

**Figure 1:**
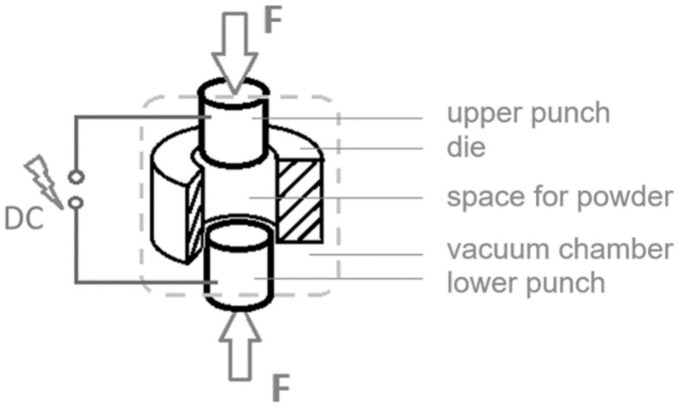
The principal structure of field assisted sintering technique (FAST) systems is a die with two moveable punches, which take the powder inside the resulting cavity. Forced pressure through the punches and temperature caused by electric resistance heating within the packed powder both consolidate the powder into a compact piece. The process set-up is normally placed in a vacuum chamber.

FAST sintering is a powder consolidation technique that uses a direct electric current and comparatively high sintering pressures to obtain powder consolidation and sintering within very short process times [11] (see Figure 1). Monchoux [12] speaks of the unprecedented speed of this process and that particularly fine microstructures are achieved by this technology, especially with regard to metals. A fine structure usually means an increase in strength. Nevertheless, its potential is not yet fully exploited.

In FAST sintering, a packed powder is heated up to sintering temperature very rapidly by resistance heating due to an electric current that flows through the die and the packed powder. Depending on the proportions of the electric resistances of the packed powder and the die, the die also heats-up and thus indirectly heats the powder. The sinter powder is then held for some minutes under forced pressure at sintering temperature. After this sintering step, the electric field is set off, and the samples are inhomogeneously cooled down to room temperature by the water cooled press rams. This cooling phase lasts several minutes, up to a quarter of an hour. During this time, the phases dissolved in the solid solution have time to change to a more relaxed state. A solid solution solidification is therefore not given.

Aluminum only became interesting as a construction material through the possibility of precipitation hardening. Along with grain refinement and work hardening, precipitation hardening is one of the ways to increase the strength of aluminum materials [13]. However, this can only be implemented with suitable alloys, because precipitation hardening requires a homogeneous solid solution at elevated temperatures and the formation of a fine-dispersed intermetallic precipitation phase during cooling, which results from decreased solubility of the alloying element(s) and supersaturation of the solid solution at lower temperatures. AlSi casting alloys can be hardened by the addition of magnesium [13]. In particular, these alloys obtain higher strength, when compared to the as-cast state, mainly due to precipitation as a result of the peak-aged state. Due to precipitation of fine secondary phases, such as Mg_2_Si, and molding of eutectic silicon, ductility properties are also improved by this hardening heat treatment [14]. AlSiMg casting alloys show the following precipitation sequence, analogously to the AlMgSi wrought alloys [14]:

α (solid solution) → cluster → GP zones → β″ → β′ → β (Mg_2_Si)

Table 1 lists selected cast Al alloys and respective heat treatments to increase strength and ductility. In each case, the heat treatment leads to improved mechanical properties, especially in the case of the underaging T64 heat treatment for AlSi7Mg alloys. The aim of the underaging T64 condition is achieving a condition before peak hardness (T6) with a shorter ageing time in order to obtain better ductility properties [15].

Other works on the heat treatment of AlSiMg alloys have investigated microstructures, optimum treatment parameters and specific alloy compositions. Birol [16] investigated the morphology of the α-Al-matrix, and whether a dendritic or globular microstructure has an influence on the response of the AlSi7Mg0.6 alloy to peak-aging T6 heat treatment. Solution annealing at 540 °C for 2 h was sufficient to completely dissolve Mg and Si in both microstructures. Peak hardness was reached after ageing for 2 h at 190 °C [16]. Emadi et al. [17] demonstrated that 4 h of solution annealing at 540 °C and 6 h of artificial ageing at 170 °C led to the highest tensile strength and elongation to failure for an A356.2 alloy. Giovanni et al. [18] investigated the influence of Cu addition on the heat treatment response of A356 cast alloy. They showed that the addition of Cu resulted in higher strength. However, without additional Cu, the ductility of A356 was up to 50% higher for underaged (155 °C, 4 h and 10 h) and for peak aged conditions (190 °C, 2 h and 4 h) after solution annealing at 530 °C for 5 h. While the longer ageing time at the lower temperature of 155 °C led to an increase in ductility, the ductility dropped by about one third after twice the ageing time at 190 °C. Pedersen and Arnberg [19] investigated the influence of solution annealing treatment on AlSiMg alloys with different Si and Mg contents. High strength was achieved after one hour of solution annealing at 540 °C, and coarse Si crystals of unmodified alloys were relatively unaffected by the solution annealing treatment. The work showed also that the ductility of aluminum alloys containing high Mg content can be increased by reducing the quenching rate. The longer diffusion time resulted in fewer vacancy clusters and in increased growth and a lower degree of coherence of β’-Mg_2_Si precipitates. However, this was at the expense of a high strength [19].

Therefore, the aim of this study was the definition of a heat treatment routine that leads to a fine and more relaxed microstructure and hence improved mechanical properties of FAST-sintered samples. To achieve high strength in combination with good ductility, an underaging heat treatment (T61) was applied.

## 2. Experimental Approach

### 2.1. Material, Sintering Process and Heat Treatment

For the present study, the powder aluminum alloy AlSi7Mg0.6, provided by ECKA Granules Germany GmbH (Velden, Germany), was used. This alloy is of high importance for several application fields due to its excellent molding ability and its good mechanical properties, such as a high strength and fracture toughness, which enables using the alloy for lightweight structural safety components. The diameter of the gas-atomized powder was <0.063 mm. Based on the method of laser diffraction, the powder alloy was analyzed three times using the particle analyzer CILAS 920 (Cilas, Orleans, France) during the determination of the powder size distribution. The average diameters d_50_, and d_10_ and d_90_, which refer to the powder sizes at 50%, 10% and 90% of the determined distribution curve, are listed in Table 2. For microstructural characterization, additional powder sections were used.

**Figure 2:**
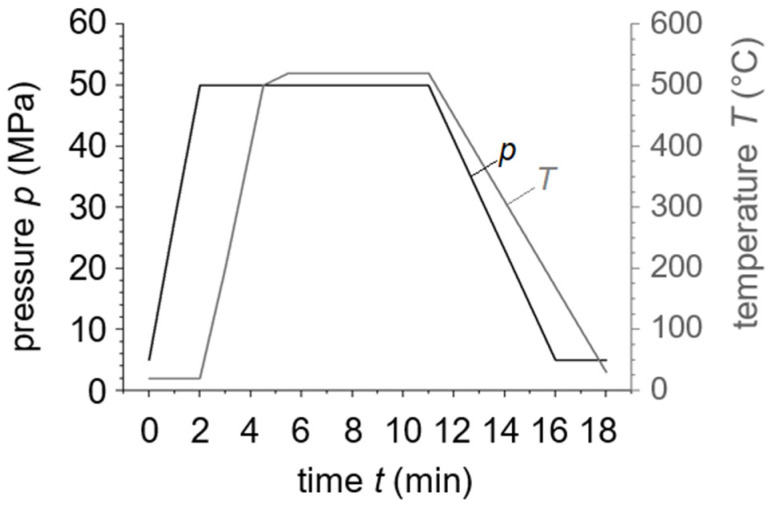
Pressure and temperature during sample production by the FAST sintering process. Powder was consolidated at 520 °C for 5 min at a pressure of 50 MPa. Before holding time, the heat rate was lowered to prevent a temperature overshoot.

From the as-received powder, six billets with a diameter of 40 mm were produced using the field assisted sintering technique (FAST) in a SPS KCE FCT-HP D 25-SI (FCT Systeme GmbH, Frankenblick, Germany). The powder was consolidated at a punch pressure of 50 MPa, a temperature of 520 °C (solid state) and a holding time of 5 min in a rough vacuum of approximately 1.2 mbar. The resistance heating was carried out by unpulsed current. After sintering samples were cooled uncontrolled using a water-cooling system on the press rams. Pressure and temperature as functions of the sintering time are shown in Figure 2.

Aiming for high ductility and strength, half of all sintered samples were heat treated. After solution annealing at 530 °C for 1 h in a convection oven and quenching in water to room temperature, the samples were subsequently artificially aged at 180 °C for 150 min to achieve a material condition with a high formability, which is referred to as condition T61 according to DIN 515 [15]. When compared to the more common condition T6 (peak-aged to maximum strength), the artificial aging time was shorter for condition T61.

### 2.2. Mechanical Properties

#### 2.2.1. Brinell Hardness

**Figure 3:**
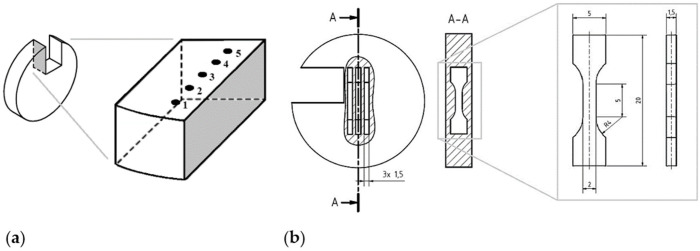
Cutting of the FAST sample for (**a**) measuring the surface Brinell hardness and the orientation of the cross section used for microscopic analysis (marked in grey), and (**b**) orientation of the extracted tensile specimens and their geometry, after [20].

To determine the Brinell hardness, five indents were executed on the surface of each FAST sample (see Figure 3a) using the hardness tester WPM LEIPZIG HPO 250 (Kögel Werkstoff- und Materialprüfsysteme GmbH, Markkleeberg, Germany). A load of 62.5 kP and a sphere with a diameter of 2.5 mm were used. To determine potential hardness differences between the inner and outer parts of the samples, the indents were executed on the middles of the samples and the edges, at equal distances from each other.

#### 2.2.2. Tensile Testing

For the investigation of the mechanical properties, three tensile specimen were eroded in the direction vertically to the applied sinter pressure for each FAST sample (see Figure 3b). For each material condition, nine samples (three specimens per sinter billet) were tested with an elongation rate of 10^−3^ s^−1^ (quasi-static) at room temperature using a Zwick-Roell Z020 universal testing machine (ZwickRoell GmbH & Co., KG, Ulm, Germany). The chosen parameters for tensile testing allowed for a quasi-static condition to avoid influences of strain rate and temperature effects. Further, the number of tested specimens provided an adequate statistical basis to ensure reproducible testing results. Elongation was measured directly on the eroded specimen surface by digital image correlation (GOM GmbH, Braunschweig, Germany) and the software IVIEW V6.3.1-2. The testXpert II (ZwickRoell GmbH & Co. KG, Ulm, Germany) software output raw data as ASCII files, which were further transferred to stress–strain data series using the software imc FAMOS Professional (IMC Test & Measurement GmbH, Berlin, Germany). For the calculation of the stress, the software FAMOS uses the manually entered thickness and width of the tensile specimen, which were previously measured and averaged three times using an outside micrometer. Evaluation of the stress–strain curves was carried out in consideration of the appendix G in DIN EN ISO 6892 [21] via an imc FAMOS script.

#### 2.2.3. Microstructural Analysis

The cross-sections used for microstructure analysis were extracted from the FAST samples as shown in Figure 3a and were prepared by standard metallographic procedures (grinding, polishing). As a last step, a polishing suspension with oxide ceramic polishing particles was used for finishing the sample surface.

The qualitative and quantitative microstructure analysis was carried out on micrographs taken by an optical microscope Olympus GX51 (Olympus Deutschland GmbH, Hamburg, Germany). The porosity was evaluated using the software Stream Motion 2.1 (Olympus Deutschland GmbH, Hamburg, Germany) via grey threshold values. Since the evaluation in the 2D range was not as significant, the density was further determined according to Archimedes principle using a density determination set, YDK01 (Sartorius, Goettingen, Germany), and a precision balance. The difference in density Δ*φ* was calculated based on the theoretically achievable density.

## 3. Results and Discussion

**Figure 4:**
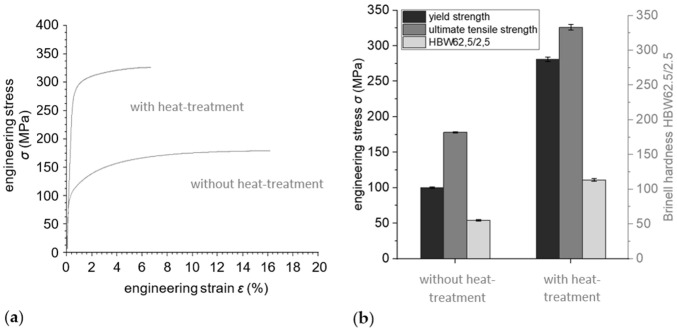
(**a**) Tensile behavior and (**b**) mechanical properties of the FAST-sintered A357 powder alloy. By heat treatment, yield and ultimate tensile strength were significantly increased, but the ductility was decreased.

**Figure 5:**
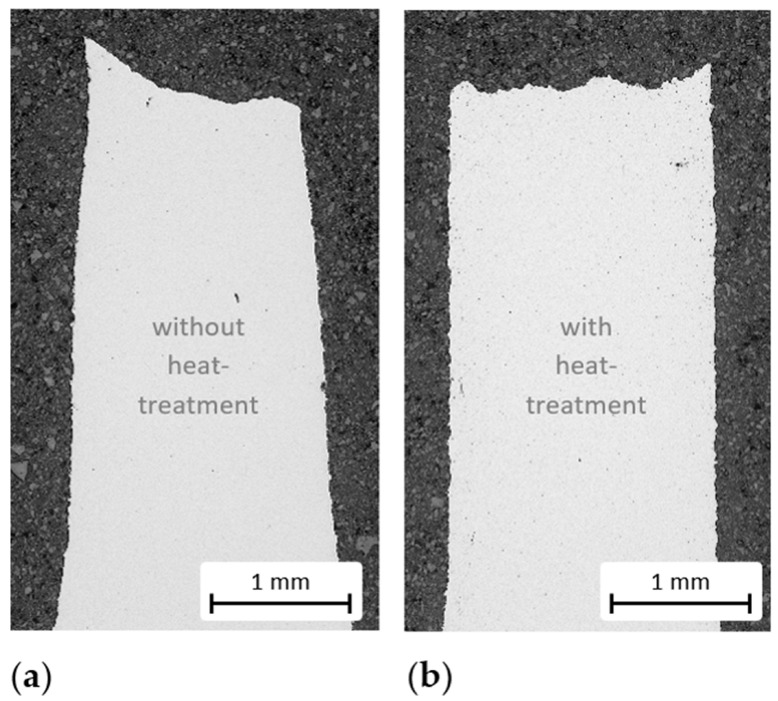
Optical micrographs of the fracture zones of the tensile tested specimens: (**a**) as-sintered and (**b**) heat-treated. The as-sintered specimens exhibited pronounced necking, which indicates higher formability in comparison to the heat treated material.

Figure 4a shows typical stress–strain curves until failure for both investigated conditions, i.e., as-sintered and heat treated. Yield and ultimate tensile strength next to the Brinell hardness are depicted in Figure 4b. Heat treatment led to an increase by nearly three times in yield strength, from (100 ± 1) MPa to (281 ± 3) MPa, and nearly doubled the ultimate tensile strength from (178 ± 1) MPa to (326 ± 4) MPa. The surface hardness increased by 100%. There were no significant differences in properties between material taken from the edges and middles of the samples. In contrast, however, the ductility of the heat-treated material was significantly reduced. The lower formability of the heat-treated condition was indicated by the more brittle fracture zone of the tested tensile specimens when compared to the as-sintered material, which showed pronounced necking (see Figure 5). However, when the mechanical properties of the heat-treated sintered material are compared to those of the as-cast alloy, according to the literature (see Table 1), the values for the yield strength are quite similar, but for the heat-treated condition the elongation to failure was halved.

**Figure 6:**
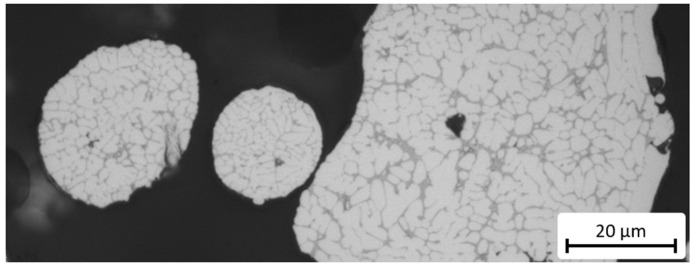
Gas-atomized powder particles of different sizes. The lower the diameter of the powder particles, the finer the dendritic microstructure. Due to their lesser volume, the cooling rate in smaller particles is higher and the crystallization is faster, which results in finer microstructures.

**Figure 7:**
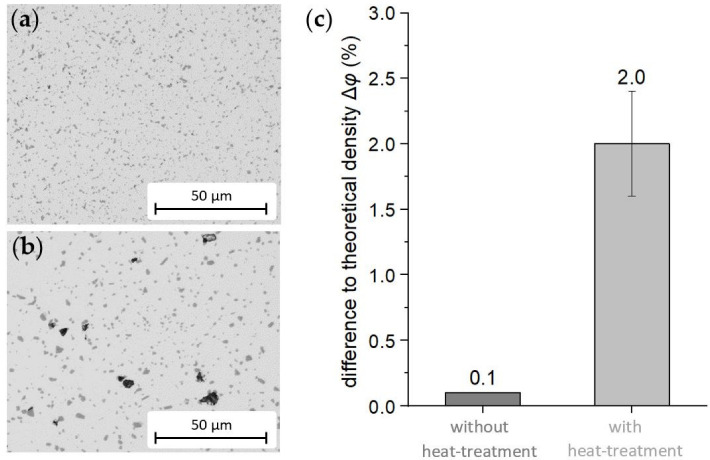
Comparison of the microstructures between (**a**) as-sintered and (**b**) heat-treated condition. (**c**) Results of density determination. The porosity was significantly higher for the heat-treated samples compared to the as-sintered material.

In Figure 6 the dendritic microstructure of the gas-atomized powder alloy is shown. During sintering, the eutectic silicon was molded to spheres less than 1 µm across (see Figure 7a). With a porosity of 0.1%, the microstructure was very dense, and the eutectic silicon was distributed regularly, which corresponds with a fine, slightly relaxed microstructural state. The microstructure after heat treatment also exhibited a regular distribution of the eutectic silicon, but its average particle size was higher, since the particle grew to up to 3 µm in diameter (see Figure 7b). This coarsening of the silicon may have been a result of the Ostwald ripening [22]. Diffusion drives down the concentrations of Si atoms when mostly large and stable Si particles are present. In contrast, the concentration of Si atoms increases when smaller, unstable particles are in proximity. Therefore, to reduce the inner energy, Si atoms diffuse toward large Si particles in the α-solid solution. These particles grow further, and smaller ones dissolve. When compared to the as-sintered condition, pronounced pores formed as a secondary result of the heat treatment.

**Figure 8:**
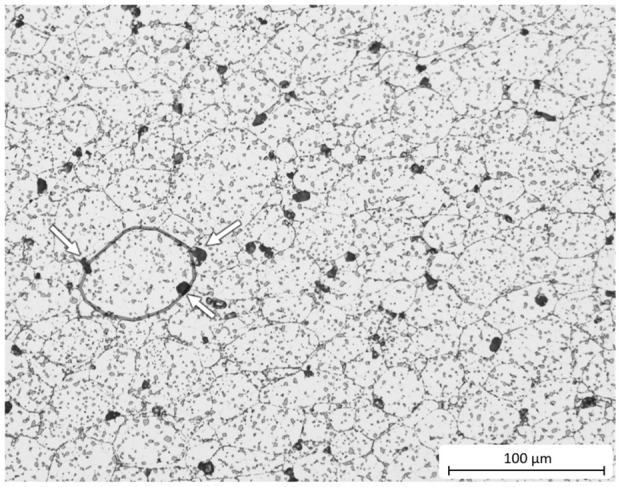
Optical micrograph of the heat-treated material etched with 10 Vol.-% hydrofluoric acid. Pores grew along the borders of former powder particles (encircled) and hence formed a network of chain-like pore paths.

By density analysis, a significant increase in porosity was measured, when comparing the heat-treated condition to the as-sintered condition (see Figure 7c). Due to the heat treatment, the porosity increased from almost zero to approximately 2%. Furthermore, similarly to the growth of eutectic silicon, small pores formed, preferably arranged in a chain along former particle boundaries (see Figure 8—a former powder particle is circled). Tammas-Williams et al. [23] also observed this phenomenon after heat treatment of additively manufactured and subsequently hot-isostatically-pressed titanium components. Due to the pronounced growth of pores during heat treatment and their large size, the porosity was further increased by the voids due to the pores resulting from the diffusion processes. Other researchers have described similar effects regarding the pore size after heat treatment of sintered, additive manufactured and cast aluminum [24,25,26].

The pronounced pores in the heat-treated material may have been a result of the oxide layer around the Al powder used for sample manufacturing due to its high oxygen affinity. During sintering, the oxide layers broke up due to the mechanical pressure. The resulting metallic contact between powder particles led to sinter necks during the sintering process. The oxygen in the aluminum alloy can also be present in atomic form and could have recombined into O_2_, thereby generating pores during the FAST process, presumably. However, during sintering, O_2_ and residual gas from the atmosphere were probably compacted under pressure in the previously undetectable pores. The temperature increase after sintering due to solution annealing resulted in an increased specific volume of the aluminum alloy without external pressure, and the gas volume in the pores increased simultaneously. A few Kelvin below solidification temperature, diffusivity increases. Defects such as voids migrate following the stress gradient towards the pressurized pore. Furthermore, voids may have arranged to clusters and subsequently into new micropores to minimize the surface energy. Oxygen continued to recombine in these pores, and its gas pressure, increased by temperature, accelerated pore growth. As a result of the equilibrium diffusion stimulated by the gradient, the internal energy of the sintered sample decreases during solution annealing. Presumably, if the samples had been cooled down sufficiently slowly after annealing, they would have retained their original volume. However, slow cooling was not performed, as for precipitation hardening, quenching after solution annealing is mandatory to enable a supersaturated solid solution and the resulting precipitation hardening. It is possible that the phenomenon of solid shrinkage, known in foundry technology, occurred during quenching from the beginning of solidification to room temperature, since shrinkage is a reduction in volume. Immediately after solution annealing, the sintered samples were cooled in water to room temperature. Due to the high cooling rates, the enlarged volume was retained as a result of the thermal expansion process, and the enlarged pores “conserved” their size. Since the sample’s mass did not change in this case (in accordance with the law of volume constancy), the theoretic density *φ* of the sample decreased.

Examples of previously undetectable two-dimensional defects include oxide skins and contact areas of original matrix powder particles, which may have grown into three-dimensional, detectable pores as a result of volume shrinkage and internal gas pressure. This additionally generated pore volume has a significantly negative influence on the mechanical properties of the sintered material. A higher number of pores can accelerate premature failure under tensile loading, as these pores act as additional voids and stress raisers.

**Figure 9:**
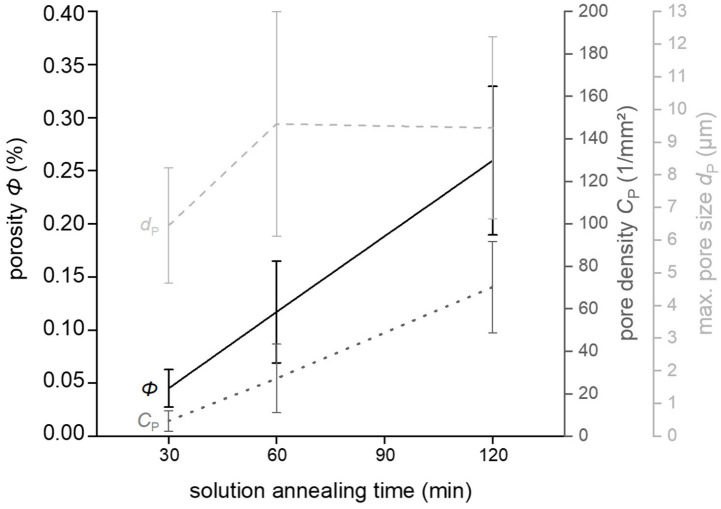
Results of quantitative microstructural analysis of porosity, pore density and maximum pore size. An increase in the solution annealing time resulted in increases in porosity, pore density and size.

To determine the cause of the porosity, further experiments were performed. The solution annealing time was 30 min, or one hour—as usual—or two hours (see Figure 9). Aging time and temperature remained unchanged (180 °C for 3 h). Cross-sections of the heat-treated samples were analyzed, and porosity and pore density, maximum pore size and equivalent circular diameters of pores were measured (compare 2.2.3). Pore density increased with increasing solution annealing time. Doubling of time resulted in a fourfold increase in pore number per mm^2^. Quadruplication of annealing time resulted in a tenfold increase in pore number. The maximum pore size showed another trend. The pore size growth stagnated after one hour of annealing. However, until this time, an increase in pore size of about one third was observed. The porosity increase due to the solution annealing time can be explained as follows: At 530 °C, the solution annealing time is close to the solidification temperature of the aluminum alloy and the diffusion movement of voids is less inhibited. In addition, there is gas pressure in the pores, as discussed above. If the solution heat treatment time, and therefore the time for equilibrium diffusion, is increased, the volume of transported material will also increase eventually. More defects can heal, void clusters can grow into micropores and further residual gas can recombine in them, increasing the pore pressure. Finally, the porosity increases.

To gain the desired mechanical properties, especially a high ductility, exact parameters for the heat treatment routine are mandatory. As presented, the heat treatment parameters for sintered materials need to be adapted to the particular requirements. A direct transfer of the heat treatment routine from that of the cast materials is not possible. The investigations in this study clearly show that the formation of pores in a sintered aluminum alloy during heat treatment is an important effect that has to be addressed. Further systematic investigations to enable an understanding of the factors influencing the pore formation and their growth is necessary. The results indicate that longer evacuation times before sintering and longer sintering times themselves could be beneficial to avoid remaining gas and former powder particle contact areas which could lead to pore growth. Despite all this, it was noticed that delay times between solution heat treatment and artificial ageing of the sintered samples resulted in smaller pores. It is possible that these effects are the results of diffusion processes influenced by natural aging or relaxation due to a decreased gas pressure in the pores, so further studies are necessary.

## 4. Conclusions

In the presented study, the effects of an underaging heat treatment T61 targeting improvements in strength and ductility for a FAST-sintered A357 aluminum alloy were investigated. The results and conclusions are summarized in the following:

1. By underaging heat treatment, yield strength was increased nearly threefold and ultimate tensile strength nearly twofold, when compared to the as-sintered material, due to resulting precipitation-hardening.
2. In contrast, the ductility was decreased by approximately two-thirds and the fracture behavior under tensile loading was more brittle for the heat-treated sintered aluminum alloy. The reason for this effect was the increased porosity after heat treatment.
3. The formation of pores and the increased porosity due to heat treatment presumably result from the temperature-driven equilibrium diffusion and increasing gas pressure inside of the micropores resulting from the sintering process, which were previously almost undetectable by optical microscopy.

## Figures and Tables

**Table 1:** Selection of aluminum casting alloys and their mechanical properties [15].

Aluminum Alloy	Heat Treatment	Yield Strength (MPa)	Ultimate Tensile Strength (MPa)	Elongation to Failure (%)	Brinell Hardness
EN-AC-AlSi12/ EN-AC-44200	F	70	150	5	50
EN-AC-AlSi9Mg/ EN-AC-43300	F T6	80 190	160 230	2 2	50 75
EN-AC-AlSi7Mg0.3/ EN-AC-42100	F T6 T64	80 190 120	140 230 200	2 2 4	50 75 60
EN-AC-AlSi7Mg0.6/ EN-AC-42200	T6	210	250	1	85
EN-AC-AlSi7Cu1Mg0.6/ EN-AC-45600	T64	250	280	3	100

**Table 2:** Powder size distribution of the as-received powder fraction.

Powder Particle Diameter	*d*_10_	*d*_50_	*d*_90_
size (µm)	22.9 ± 0.1	40.3 ± 0.1	63.7 ± 0.8

## Data Availability

The authors confirm that the data to support the findings of this study are available within the article.
